# Taylor Series Approximation for Accurate Generalized Confidence Intervals of Ratios of Log‐Normal Standard Deviations for Meta‐Analysis Using Means and Standard Deviations in Time Scale

**DOI:** 10.1002/pst.2467

**Published:** 2025-01-23

**Authors:** Pei‐Fu Chen, Franklin Dexter

**Affiliations:** ^1^ Department of Anesthesiology Far Eastern Memorial Hospital New Taipei City Taiwan; ^2^ Department of Electrical Engineering Yuan Ze University Taoyuan Taiwan; ^3^ Department of Anesthesia University of Iowa Iowa City Iowa USA

**Keywords:** anesthesia time, extubation time, generalized confidence intervals, log‐normal distribution, surgical time

## Abstract

With contemporary anesthetic drugs, the efficacy of general anesthesia is assured. Health‐economic and clinical objectives are related to reductions in the variability in dosing, variability in recovery, etc. Consequently, meta‐analyses for anesthesiology research would benefit from quantification of ratios of standard deviations of log‐normally distributed variables (e.g., surgical duration). Generalized confidence intervals can be used, once sample means and standard deviations in the raw, time, scale, for each study and group have been used to estimate the mean and standard deviation of the logarithms of the times (i.e., “log‐scale”). We examine the matching of the first two moments versus also using higher‐order terms, following Higgins et al. 2008 and Friedrich et al. 2012. Monte Carlo simulations revealed that using the first two moments 95% confidence intervals had coverage 92%–95%, with small bias. Use of higher‐order moments worsened confidence interval coverage for the log ratios, especially for coefficients of variation in the time scale of 50% and for larger $\left(n=50\right)$ sample sizes per group, resulting in 88% coverage. We recommend that for calculating confidence intervals for ratios of standard deviations based on generalized pivotal quantities and log‐normal distributions, when relying on transformation of sample statistics from time to log scale, use the first two moments, not the higher order terms.

## 1 Introduction

Contemporary anesthetic drugs assure the efficacy of general anesthesia. Multiple new drugs have achieved global regulatory approval, and yet little adoption. The recurring pattern is that following approval in each country (region), quickly multiple appropriately sized randomized trials (i.e., 15–50 patients per group) [1] compare outcomes such as time to awakening between the new drug and one of the several decades‐old generic products. Medians and means differ significantly but not enough to impactful clinically or managerially [2]. Yet, multiple industrial engineering type studies show economic and perceived benefits of the newer anesthetics from reductions in variability in dosing, variability in recovery, etc. [2].

Realistically, for anesthesia drugs and techniques (e.g., use of regional anesthesia with adjuvant agents), health‐economic arguments will continue to be addressed after drug approval and a few years of use. Practically, anesthesia practitioners care for patients undergoing so many different surgical procedures [3] that many randomized trials, most with approximately 25 patients per group, will continue to be the process of drug roll‐out. Our focus is to improve upon the meta‐analysis of these studies for use in health economic comparisons [2, 4].

Comparisons between groups of variability in times to recovery, specifically ratios of standard deviations, can be analyzed using generalized pivotal methods based on the log normal [4, 5, 6]. Ratios are compared because the surgical procedures are an extremely important covariate, but are nearly always the same in both groups, such that precision of the ratios is much larger than among differences [4]. The method to compare ratios of standard deviations is simple to perform, with just a few lines of Stata, R, SAS, Python, etc., code, or Office 365 Excel with its dynamic arrays [4]. The anesthesia times, patient recovery times, etc., often follow log‐normal distributions [7, 8, 9, 10, 11, 12, 13]. However, for application to meta‐analysis, without the raw data, first the means and standard deviations in the log scale need to be estimated from the means and standard deviations in the time scale [4, 6, 14, 15]. In other words, the means and standard deviations of the logarithms of the times to recovery (“log scale”) need to be obtained from the sample means and standard deviations of the times to recovery in minutes or hours (i.e., “time scale”).

The goal of our article is to evaluate the performance of methods to estimate the standard deviations in the log scale from the statistics in the time scale. Higgins et al. examined the use of higher order moments to enhance accuracy of transformation from raw to log scale [14]. Based on Friedrich's subsequent simulations, we hypothesized performance not exceeding that achieved by utilizing the first two moments for estimation [15]. However, those simulations were not for modeling the ratios of standard deviations [15]. We used Monte‐Carlo simulation to combine generalized confidence intervals for ratios of standard deviations [4, 5] with Higgins' comparison [14] of the matching the first two moments of the log‐normal versus the use of higher‐order moments. We aim to learn if confidence interval coverage is significantly better with using the first two moments, the first two and higher‐order moments, or makes no difference.

## 2 Methods

Durations in the time scale are $x_{i,j,p}$, where i represents the treatment group (*i* = 1 and *i* = 2), j the study (j = 1, …, g), and p the patient within the study (p = 1, …, $n_{i,j}$). Based on Scopus search, we considered sample sizes *n* = 15, *n* = 25, and *n* = 50, and coefficients of variation *CV* = 15%, *CV* = 30%, and *CV* = 50% (see Data S1) [4, 16, 17]. For variable X with a log‐normal distribution, $Z=\ln\left(X\right)\sim N\left(\mu ,\sigma_{z}^{2}\right).$ Letting $\mu_{i}$ and $\sigma_{z_{i}}$ represent the mean and standard deviation of log‐transformed measurements: [4, 9, 14]

(1) $$\sigma_{z_{i}}=\text{sqrt}\left(\ln\left(1+{{\mathrm{CV}}_{x_{i}}}^{2}\right)\right)$$

where ${\mathrm{CV}}_{x_{i}}$ denotes the coefficient of variation of the durations in the time scale in the *i*th group.

There is currently one described method for calculating confidence intervals for ratios of the log‐normal standard deviations, a computational method that takes a second or two [4, 5]. For each simulation iteration, with $\mu_{1}=\mu_{2}$ = 1 and $\sigma_{1}=\sigma_{2}$ we generated $n_{1}+n_{2}$ log‐normally distributed samples. These two groups' samples will be compared. Using the sampled mean (${\bar{z}}_{i}$) and standard deviation (${s_{z}}_{i}$) in the log scale from these two groups, the generalized pivot statistics are calculated for the log ratio of the standard deviation (δ): [4]

(2) $${Τ}_{\delta }^{k}=\ln\left(T_{\mu_{1}}^{k}\right)-\ln\left(T_{\mu_{2}}^{k}\right)$$

where

(3) $$\begin{array}{cc} T_{\mu_{i}}^{k} & =\exp\left\{{\bar{z}}_{i}-{\left[\frac{{{s_{z}}_{i}}^{2}\left(n_{i}-1\right)}{U^{k}}\right]}^{\frac{1}{2}}∙\frac{Z^{k}}{\sqrt{n_{i}}}\right\}\\ & ∙{\left\{\left[\exp\left(\frac{{{s_{z}}_{i}}^{2}\left(n_{i}-1\right)}{U^{k}}\right)-1\right]\exp\left[\frac{{{s_{z}}_{i}}^{2}\left(n_{i}-1\right)}{U^{k}}\right]\right\}}^{1/2} \end{array}$$

and for *k* = 1 to *m* iterations,

**Table:** 

Generate $U^{k}$	a chi‐square distributed random number with $n_{i}$ – 1 degrees of freedom
Generate $Z^{k}$	a normally distributed random number with mean 0 and variance 1

Equation (3) is a variation on the algebra inherent to Student's *t*‐test [18]. There are the terms including $U^{k}$ because $\left[\left(n_{\mathrm{ij}}-1\right){\hat{\sigma }}_{\mathrm{ij}}^{2}/\sigma_{\mathrm{ij}}^{2}\right]$ follows a chi‐square distribution with *n*_*ij*_−1 degrees of freedom [18, 19]. There is a term including $Z^{k}$ because $\left[\sqrt{n_{\mathrm{ij}}\;}\left({\hat{\mu }}_{\mathrm{ij}}-\mu_{\mathrm{ij}}\right)/\sigma_{\mathrm{ij}}\right]$ follows a standard normal distribution [18, 19].

Continuing, using *m* = 99,999: [4]

(4) $$\ln\left(\text{ratio}\right)=\text{median of}\;{Τ}_{\delta }^{k}$$

and

(5) $$\mathrm{SE}\;\text{of}\;\ln\left(\text{ratio}\right)=\frac{75\mathrm{th}\;\text{percentile of}\;{Τ}_{\delta }^{k}-25\mathrm{th}\;\text{percentile of}\;{Τ}_{\delta }^{k}}{\Phi^{-1}\left(0.75\right)-\Phi^{-1}\left(0.25\right)}$$

where $\Phi^{-1}$ represents the inverse of the standard normal cumulative distribution function. The choice of 99,999 lets the quantiles used (50th, 25th, 75th) be order statistics [4, 6]. The quantiles are used because the simulations generate some very small and large outliers [4, 6].

Confidence limits in the log scale are:

(6) $$\ln\left(\text{ratio}\right)\pm \Phi^{-1}\left(1-\frac{\alpha }{2}\right)*\mathrm{SE}\;\text{of}\;\ln\left(\text{ratio}\right)$$

Using α = 0.05 and *g* = 100,000 Monte‐Carlo simulations, equation (6) should include the true *ln*(*ratio*) = 0 approximately 95% (Table 1). Computer code is in Data S1.

**TABLE 1:** Generalized confidence intervals for the ratio of two standard deviations calculated from the simulated data in the log scale, both groups having the same log‐normal distributions.^a^

*n*_1_ = *n*_2_	*CV*_1_ = *CV*_2_ ^b^	% Coverage^c^	Mean of bias^d^	SE of bias^e^	% simulations with abs (ln(*ratio*)) > 0.5^f^
15	0.15	94.7	0.00262	0.00029	8.6
	0.3	95.1	0.00284	0.00033	12.9
	0.5	95.5	0.00342	0.00041	21.8
25	0.15	94.9	0.00177	0.00022	2.3
	0.3	95.1	0.00187	0.00025	4.5
	0.5	95.4	0.00223	0.00031	10.5
50	0.15	95.0	0.00106	0.00015	0.1
	0.3	95.1	0.00110	0.00017	0.4
	0.5	95.2	0.00132	0.00021	2.0
*n*_1_ = 15, *n*_2_ = 50	0.15	94.9	0.00755	0.00023	3.2
	0.3	95.0	0.00682	0.00026	5.8
	0.5	95.1	0.00345	0.00033	12.4

Abbreviations: *CV*, coefficient of variation; *n*, number of cases; SE, standard error.

^a^ Results presented are based on 1 million simulations.

^b^ All means in the log scale equaled 1.0. For *CV* = 0.15, 0.30, and 0.50, the standard deviations in the log scale equaled 0.15, 0.29, and 0.47, respectively. The means in the time scale were 2.75, 2.84, and 3.04, respectively. The standard deviations in the time scale were 0.41, 0.85, and 1.52, respectively.

^c^ The percentage of the 95% confidence intervals that include 0 for ln(ratio), ideally equaling nominal 95%. The nine standard errors of the percentages were all ≤ 0.02%.

^d^ The arithmetic mean of the *ln*(*ratio*) – 0 for all simulations.

^e^ The standard deviation of the ln(ratio) divided by the square root of 1,000,000.

^f^ The distributions are symmetric, representing the difference of randomly generated values. The thresholds of ± 0.5 in the log scale correspond to ratios of standard deviations of 0.61 and 1.65, respectively.

For meta‐analysis without raw data, sample means $\left({\bar{x}}_{i}\right)$ and standard deviations $\left(s_{x_{i}}\right)$ in the time scale are transformed to log scale. We compared two methods: matching the first two moments (indicated by single dashes on ${{\bar{z}}_{i}}^{\prime }$ and ${s_{z_{i}}}^{\prime }$) (Table 2) and the higher‐order terms from Higgins' et al. Taylor series approximation (indicated by double dashes on ${{\bar{z}}_{i}}^{\prime \prime }$ and ${s_{z_{i}}}^{\prime \prime }$) (Table 3) [14]. We used Friedrich's reparameterization [15]. The logarithmic mean: [14, 15]

(7) $${{\bar{z}}_{i}}^{\prime }=\ln\left(\frac{{\bar{x}}_{i}}{\sqrt{1+{{\mathrm{CV}}_{x_{i}}}^{2}}}\right)$$

**TABLE 2:** Generalized confidence intervals for the ratio of two standard deviations applying the first two moments to convert sample means and standard deviations in the time scale to the log scale.^a^

*n*_1_ = *n*_2_	*CV*_1_ = *CV*_2_ ^b^	% Coverage^c^	Mean of bias^d^	SE of bias^e^	% simulations with abs (ln(*ratio*)) > 0.5^f^
15	0.15	94.5	0.00264	0.00029	8.9
	0.3	94.6	0.00287	0.00034	13.8
	0.5	94.7	0.00340	0.00042	23.1
25	0.15	94.6	0.00176	0.00022	2.5
	0.3	94.1	0.00185	0.00026	5.6
	0.5	93.5	0.00218	0.00033	13.2
50	0.15	94.5	0.00105	0.00016	0.1
	0.3	93.4	0.00110	0.00019	0.8
	0.5	92.0	0.00130	0.00024	4.2
*n*_1_ = 15, *n*_2_ = 50	0.15	94.6	0.01070	0.00023	3.4
	0.3	94.1	0.01917	0.00027	6.7
	0.5	93.3	0.03643	0.00034	14.6

Abbreviations: *CV*, coefficient of variation; *n*, number of cases; SE, standard error.

^a^ Results presented are based on 1 million simulations.

^b^ All means in the log scale equaled 1.0. For *CV* = 0.15, 0.30, and 0.50, the standard deviations in the log scale equaled 0.15, 0.29, and 0.47, respectively. The means in the time scale were 2.75, 2.84, and 3.04, respectively. The standard deviations in the time scale were 0.41, 0.85, and 1.52, respectively.

^c^ The percentage of the 95% confidence intervals that include 0 for ln(ratio), ideally equaling nominal 95%. The nine standard errors of the percentages were all < 0.03%.

^d^ The arithmetic mean of the *ln*(*ratio*) – 0 for all simulations.

^e^ The standard deviation of the ln(ratio) divided by the square root of 1,000,000.

^f^ The distributions are symmetric, representing the difference of randomly generated values. The thresholds of ± 0.5 in the log scale correspond to ratios of standard deviations of 0.61 and 1.65, respectively.

**TABLE 3:** Generalized confidence intervals for the ratio of two standard deviations applying the higher order of moments to convert sample means and standard deviations in the time scale to the log scale.^a^

*n*_1_ = *n*_2_	*CV*_1_ = *CV*_2_ ^b^	% Coverage^c^	Mean of bias^d^	SE of bias^e^	% simulations with abs (ln(*ratio*)) > 0.5^f^
15	0.15	94.5	0.00264	0.00029	8.9
	0.3	94.3	0.00288	0.00034	14.3
	0.5	91.9	0.00345	0.00054	28.8
25	0.15	94.5	0.00176	0.00022	2.6
	0.3	93.7	0.00185	0.00026	5.9
	0.5	90.1	0.00227	0.00042	18.7
50	0.15	94.4	0.00105	0.00016	0.1
	0.3	93.0	0.00110	0.00019	0.9
	0.5	88.2	0.00130	0.00029	7.8
*n*_1_ = 15, *n*_2_ = 50	0.15	94.6	0.01068	0.00024	3.5
	0.3	93.8	0.01860	0.00028	7.1
	0.5	90.3	0.02624	0.00043	20.0

Abbreviations: *CV*, coefficient of variation; *n*, number of cases; SE, standard error.

^a^ Results presented are based on 1 million simulations.

^b^ All means in the log scale equaled 1.0. For *CV* = 0.15, 0.30, and 0.50, the standard deviations in the log scale equaled 0.15, 0.29, and 0.47, respectively. The means in the time scale were 2.75, 2.84, and 3.04, respectively. The standard deviations in the time scale were 0.41, 0.85, and 1.52, respectively.

^c^ The percentage of the 95% confidence intervals that include 0 for ln(ratio), ideally equaling nominal 95%. The nine standard errors of the percentages were all ≤ 0.03%.

^d^ The arithmetic mean of the *ln*(*ratio*) – 0 for all simulations.

^e^ The standard deviation of the ln(ratio) divided by the square root of 1,000,000.

^f^ The distributions are symmetric, representing the difference of randomly generated values. The thresholds of ± 0.5 in the log scale correspond to ratios of standard deviations of 0.61 and 1.65, respectively.

The logarithmic standard deviation: [14, 15]

(8) $${s_{z_{i}}}^{\prime }=\sqrt{\ln\left(1+{{\mathrm{CV}}_{x_{i}}}^{2}\right)}$$

Using the higher order terms: [15]

(9) $$\begin{array}{cc} {s_{z_{i}}^{2}}^{\prime \prime } & ={{\mathrm{CV}}_{x_{i}}}^{2}{\left(1+\frac{{{\mathrm{CV}}_{x_{i}}}^{2}}{1+{{\mathrm{CV}}_{x_{i}}}^{2}}\right)}^{2}-\left(1+\frac{{{\mathrm{CV}}_{x,i}}^{2}}{1+{{\mathrm{CV}}_{x_{i}}}^{2}}\right)\left[{\left(1+{{\mathrm{CV}}_{x_{i}}}^{2}\right)}^{2}-3+\frac{2}{1+{{\mathrm{CV}}_{x_{i}}}^{2}}\right]\\ & +\frac{1}{4}\left[{\left(1+{{\mathrm{CV}}_{x_{i}}}^{2}\right)}^{4}-4\left(1+{{\mathrm{CV}}_{x_{i}}}^{2}\right)-1+\frac{8}{1+{{\mathrm{CV}}_{x_{i}}}^{2}}-\frac{4}{{\left(1+{{\mathrm{CV}}_{x_{i}}}^{2}\right)}^{2}}\right] \end{array}$$

From Friedrich et al., for larger coefficients of variation (e.g., *CV* = 0.5), the third term in the equation becomes dominant, resulting in an increase in the variance, ${s_{z_{i}}}^{\prime \prime }$ [15]. The log‐transformed mean (${{\bar{z}}_{i}}^{\prime }$) and standard deviation (${s_{z_{i}}}^{\prime }$ and ${s_{z_{i}}}^{\prime \prime }$) were then used in Equations (2), (3), (4), (5), and (6). These calculations were repeated for *g* = 1,000,000 to estimate the percentage coverage and mean bias (Tables 2 and 3). Because the simulations satisfy the null hypothesis, they are the same as 10,000 simulations of meta‐analysis with 10 two‐group studies.

## 3 Results

To check validity of our Monte Carlo simulations including the use of equations (4) and (5), generalized confidence intervals were calculated using simulated data in the log scale. Sample sizes (*n*) were 15, 25, or 50, and coefficient of variations in the time scale were 0.15, 0.3, or 0.5 [1, 8, 9]. Type I error rates for nominal 95% confidence intervals ranged from 94.7% to 95.5% (Table 1). Further confirming validity, as expected analytically [19], when *n* was reduced to 2, percentage coverages were less, 92.6% for a *CV* of 0.15.

We simulated the use of reported sample means and standard deviations in the time scale by the matching of the first two moments equations (7) and (8). Percentage coverages ranged from 92.0% to 94.7% (Table 2). Coverage decreased from nominal as *n* increased. For example, for *CV* = 0.5, 94.7% for *n* = 15 versus 92.0% for *n* = 50. Biases were small, as useful for meta‐analyses.

Our primary objective was to quantify the impact of Higgins et al.'s derived higher‐order moments for the transformation of means and standard deviations from the time scale to the log scale [14, 15]. Percentage coverages were less accurate pairwise (Tables 2 and 3), 88.2%–94.5%. The accuracy decreased when *CV* increased and *n* increased. For example, for *n* = 50, 94.4% for *CV* = 0.15 versus 88.2% for *CV* = 0.5. When *n* was increased to 100, percentage coverage for *CV* = 0.5 reduced further, 86.8%. The relationship was the same when *n*_1_ = 15 (smallest) and *n*_2_ = 50 (largest) (Tables 2 and 3).

Examining Tables 1, 2, 3 pairwise by row, progressively percentage coverages were less (3rd columns), Table 3 less accurate than Table 2 than Table 1. The percentages of simulated ratios < 0.61 or > 1.65 (6th column) increased. For example, the largest percentage $\ln\left(\text{ratio}\right)$ in the tails was for the smallest n and largest CV, 21.8% in Table 1. That percentage increased to 23.1% in Table 2 and 28.8% in Table 3, although there was no greater population variance.

## 4 Discussion

When generalized confidence intervals were used to estimate ratios of standard deviations of log‐normal distributions, and the sample estimates were from the log scale, 95% confidence intervals had nominal coverage, without bias. When data were limited to means and standard deviations in the time scale, accurate 95% confidence intervals were obtained neither using first two moments (equation 8) nor higher order moments (equation 9). However, the bias obtained when using the simpler equation (8) was small, such that the pooled bias would be negligible for meta‐analyses. That was the approach used in the recent meta‐analysis showing its application with “real‐world” data [17]. The confidence interval coverage was further from nominal when including the higher order moments, particularly for larger *CV* (Tables 2 and 3). As explained in the Methods, Friedrich et al. showed previously that the third term of equation (9) becomes progressively larger with greater *CV*, resulting in ${s_{z_{i}}}^{\prime \prime }>{s_{z_{i}}}^{\prime }$. We recommend that for the inevitable [3] meta‐analyses of recovery times in anesthesia (e.g., for economic assessments) [2, 4, 6, 8, 16, 17], use the first‐two moments.

## Author Contributions

Dr. Chen helped with software, formal analysis, investigation, resources, data curation, writing original draft, and writing review and editing. Dr. Dexter helped with conceptualization, methodology, validation, writing original draft, writing review and editing, and visualization.

## Conflicts of Interest

The authors declare no conflicts of interest.

## Supporting information


**Data S1.** Supporting Information.

## Data Availability

The data that supports the findings of this study are available in the Data S1 of this article.
