# Supplementary material for: Taylor Series Approximation for Accurate Generalized Confidence Intervals of Ratios of Log‐Normal Standard Deviations for Meta‐Analysis Using Means and Standard Deviations in Time Scale
Source: Pharm Stat. 2025 Jan 23;24(1):e2467. doi: 10.1002/pst.2467 (PMC11755222; doi:10.1002/pst.2467)
Supplement: Supplementary file 1 — Data S1. Supporting Information. [file PST-24-0-s001.pdf]

Table of contents

Page	Section
1	Summary of sample sizes and coefficients of variation
3	Python computer code for Tables 1-3, presented without multithread computation. Results are same as those of multithread but executes slowly.<br>This computer code is easier to read. The code is online at GitHub:<br>https://FDshort.com/Chen_PST1
6	Python computer code for Tables 1-3. The code is online:<br>https://FDshort.com/Chen_PST2

Scopus search was performed (i.e., updated) 9 September 2023:

TITLE ("time to extubation" OR "times to extubation" OR "time to tracheal extubation" OR "times to tracheal extubation" OR "extubation time" OR "extubation times")

AND

TITLE ("meta-analysis")

AND

NOT TITLE (protocol OR protocols)

These 7 publications had 12 meta-analyses. Among the 12 meta-analyses, the median number of studies was 25, the 25th percentile was 20, and 75th percentile was 38. Among the 11 of these 12 meta-analyses published in an anesthesia journal, the smallest sample size of studies in the meta-analysis was 13 studies. The other had 4 studies. Therefore, in the last paragraph of the Results we used 10 studies as the smallest characteristic for these data in anesthesia. Meta-analyses of surgical times in anesthesia characteristically include scores of studies, and there have not been multiple meta-analyses for time until ready for phase I post-anesthesia care unit discharge[8].

Among the 7 publications, the one including the most studies was just published, Reference (12). That paper's supplemental Tables 3 and 4 were used for calculation. There were 78 studies, thus supplying 156 observations, where 156 = 78 x 2 groups. Among the 156 observations, the sample sizes of patients per group were median n=26, 25th percentile n=20, and 75th percentile n=35. For simulations in all paragraphs of the Results, we used sample sizes of 25 for typical, 15 for small because less than the 25th percentile, and 50 for large because larger than the 75th percentile. Among the 156 observations, the observed coefficients of variation were median CV=39%, 25th percentile CV=29%, and 75th percentile CV=53%. For simulations in all paragraphs of the Results, we used 30%, 15%, and 50%. The coefficients of variation for surgical times, anesthesia times, and readiness for post-anesthesia care unit discharge times are comparable.[4,6-8]

```
#There are two code files. This one, using no multithread, runs slowly, but easier to read and obtains results identical to at least 16 digits as the multithread used for the simulations.
import pandas as pd
import numpy as np
from scipy.stats import norm, chi2
from datetime import datetime
from math import sqrt, log, exp


def Pivot_calculation(rSampleMeanLogScale, rSampleSDLogScale, N, U, Z):
    # Equation 3
    return np.exp(rSampleMeanLogScale- np.sqrt((rSampleSDLogScale**2 * (N-1))/U) * (Z/sqrt(N)) ) * np.sqrt((np.exp((rSampleSDLogScale**2 * (N-1))/U) - 1) *
np.exp((rSampleSDLogScale**2 * (N-1))/U))

def transform_from_raw_to_log_mean_SD(Mean, SD):
    CV = SD/Mean
    CVsq = CV**2
    #Mean in log scale, Equation 7
    MeanLogScale_1 = log(Mean/sqrt(CVsq + 1))
    #SD in log scale, Equation 8
    SDLogScale_1 = sqrt(log((CVsq + 1)))
    SDLogScale_2 = sqrt(CVsq * (1 + CVsq/(1 + CVsq))**2 - (1 + CVsq/(1 + CVsq)) * ((1 + CVsq)**2 - 3 + 2/(1 + CVsq)) + (1/4) * ((1 + CVsq)**4 - 4*(1 + CVsq) - 1 + 8/(1 + CVsq) -
4/((1 + CVsq)**2)))
    #SD in log scale, Equation 9
    return MeanLogScale_1, SDLogScale_1, SDLogScale_2


# number of Monte Carlo Simulations
nMonte = 1000000

# Calculate z-score for alpha = 0.05
# ppf is the percent point function that is inverse of cumulative distribution function
z_score = norm.ppf(1 - 0.05 / 2)

# the number for pivot, the notation "m" in the manuscript
nSimulForPivot = 100000-1

# choosing a seed
seed_value = 12181988

# Generate 4 set of random numbers each with specified seed, will be used for U1, U2, Z1, and Z2 later (Equation 3)
np.random.seed(seed_value - 1)
random_numbers1_1 = np.random.rand(nSimulForPivot)

np.random.seed(seed_value - 2)
random_numbers1_2 = np.random.rand(nSimulForPivot)

np.random.seed(seed_value - 3)
random_numbers2_1 = np.random.rand(nSimulForPivot)

np.random.seed(seed_value - 4)
random_numbers2_2 = np.random.rand(nSimulForPivot)

# for Table 1, 2, and 3, respectively
for method_of_moments in ['no_moments', 'first_two_moment', 'higher_orders_of_moments']:

    # Sample size, we choose 15, 25, 50, notation "n" in the manuscript
    for N in [15, 25, 50]:
        N1 = N
        N2 = N1

        for CV in [0.15, 0.3, 0.5]:
            # coefficient of variation, we choose 0.15, 0.3, 0.5
            CV1 = CV
            CV2 = CV1

            # Mean in log scale, notation "µ_i" in the manuscript
            rMeanLogScale1 = 1
```

```
rMeanLogScale2 = rMeanLogScale1

# Standard deviation in log scale, notation "σ_i" in the manuscript
rSDLogScale1 = sqrt(log(1 + CV1 ** 2))  #Equation 1 in the manuscript
rSDLogScale2 = rSDLogScale1

# Generate random number for later used in calculating Ui and Zi in generalized pivotal method
# group 1 pivot calculation
# U_i and Z_i used in Equation 3
U1 = chi2.ppf(random_numbers1_1, N1 - 1 )
Z1 = norm.ppf(random_numbers2_1)

#group 2 pivot calculation
# U_i and Z_i used in Equation 3
U2 = chi2.ppf(random_numbers1_2, N2 - 1 )
Z2 = norm.ppf(random_numbers2_2)

#collecting results
dict_results = {'ln_ratio': [], 'se_ln_ratio': [], 'coverage': []}
# the pre-determined list of seeds, using number of nMonte
list_seeds = [i for i in range(seed_value, seed_value + nMonte)]
for seed_ in list_seeds:
    # Calculate the mean and standard deviation of a sample generated from a random generator of a normal distribution
    np.random.seed(seed_)
    # generate log-normal distribution, using mean of rMeanLogScale and standard deviation of rSDLogScale
    rSampleOfRandoms = [norm.ppf(i,loc=rMeanLogScale1, scale=rSDLogScale1) for i in np.random.rand(N1+N2)]
    #using no method of moments
    if method_of_moments == 'no_moments':
        rSampleOfRandoms1 = rSampleOfRandoms[:N1]
        rSampleOfRandoms2 = rSampleOfRandoms[N1:N1+N2]
        # the mean of rSampleOfRandoms1, notation "z_i"
        rSampleMeanLogScale1 = np.mean(rSampleOfRandoms1)
        # the standard deviation of rSampleOfRandoms1, delta degree of freeden = 1, notation "sz_i"
        rSampleSDLogScale1 = np.std(rSampleOfRandoms1, ddof=1)
        rSampleMeanLogScale2 = np.mean(rSampleOfRandoms2)
        rSampleSDLogScale2 = np.std(rSampleOfRandoms2, ddof=1)

    # using method of moments to transform from raw to log mean and SD
    else:
        rSampleOfRandoms = np.exp(rSampleOfRandoms)
        rSampleOfRandoms1 = rSampleOfRandoms[:N1]
        rSampleOfRandoms2 = rSampleOfRandoms[N1:N1+N2]

        rSampleMeanTimeScale1 = np.mean(rSampleOfRandoms1)
        rSampleSDTimeScale1 = np.std(rSampleOfRandoms1, ddof=1)
        rSampleMeanTimeScale2 = np.mean(rSampleOfRandoms2)
        rSampleSDTimeScale2 = np.std(rSampleOfRandoms2, ddof=1)

        #using Equation 7 and 8
        if method_of_moments == 'first_two_moment':
            rSampleMeanLogScale1, rSampleSDLogScale1, _ = transform_from_raw_to_log_mean_SD(rSampleMeanTimeScale1, rSampleSDTimeScale1)
            rSampleMeanLogScale2, rSampleSDLogScale2, _ = transform_from_raw_to_log_mean_SD(rSampleMeanTimeScale2, rSampleSDTimeScale2)

        #using Equation 7 and 9
        elif method_of_moments == 'higher_orders_of_moments':
            rSampleMeanLogScale1, _, rSampleSDLogScale1, = transform_from_raw_to_log_mean_SD(rSampleMeanTimeScale1, rSampleSDTimeScale1)
            rSampleMeanLogScale2, _, rSampleSDLogScale2, = transform_from_raw_to_log_mean_SD(rSampleMeanTimeScale2, rSampleSDTimeScale2)

    # Equation 3
    Pivot1 = Pivot_calculation(rSampleMeanLogScale1, rSampleSDLogScale1, N1, U1, Z1)
    Pivot2 = Pivot_calculation(rSampleMeanLogScale2, rSampleSDLogScale2, N2, U2, Z2)

    # Equation 2, generalized pivotal statistics
    pivot_statistics = np.log(Pivot1) - np.log(Pivot2)

    # Calculate ln ratio and SE ln ratio by percentile and Z statistics
    ln_ratio = pd.Series(pivot_statistics).quantile(.5)
    se_ln_ratio = (pd.Series(pivot_statistics).quantile(.75) - pd.Series(pivot_statistics).quantile(.25))/(norm.ppf(.75) - norm.ppf(.25))
```

```
    # Calculate the confidence intervals with z_score
    lower_bound = ln_ratio - z_score * se_ln_ratio
    upper_bound = ln_ratio + z_score * se_ln_ratio

    dict_results['ln_ratio'].append(ln_ratio)
    dict_results['se_ln_ratio'].append(se_ln_ratio)
    dict_results['coverage'].append((lower_bound < 0) and (upper_bound > 0))

end_time = datetime.now()

# print out the percentage of coverage
print(f'MoM={method_of_moments} N={N1} CV={CV1} percentage coverage: {np.mean(dict_results["coverage"])}')

output_dir = f"GPM_MC_nMonte_{nMonte}_N_{N1}_CV_{CV1}_{str(end_time).split('.')[0].replace('-','').replace(' ','').replace(':','')}"
print('csv save to ' + output_dir + f'_{method_of_moments}.csv')

# save the results to the csv
pd.DataFrame(dict_results).to_csv(output_dir + f'_{method_of_moments}.csv')
```

```
#This one, using the multithread, runs quickly (about 100 min for each 1,000,000 simulations). It obtains results identical to at least 16 digits as the one using no multithread.
import pandas as pd
import numpy as np
from scipy.stats import norm, chi2
from datetime import datetime
from math import sqrt, log, exp
import dask.dataframe as dd

class SimulPivotMC(object):
    def __init__(self, nMonteSim, N, CV):
        # number of Monte Carlo Simulation
        self.nMonte = nMonteSim

        # Calculate z-score for alpha = 0.05, ppf is the percent point function that is inverse of cumulative distribution function
        self.z_score = norm.ppf(1 - 0.05 / 2)

        # Sample size, we choose 15, 25, 50, notation "n" in the manuscript
        self.N1 = N
        self.N2 = self.N1

        # coefficient of variation, we choose 0.15, 0.3, 0.5
        self.CV1 = CV
        self.CV2 = self.CV1

        # Mean in log scale, notation "µ_i" in the manuscript
        self.rMeanLogScale1 = 1
        self.rMeanLogScale2 = self.rMeanLogScale1

        # Standard deviation in log scale, notation "σ_i" in the manuscript, Equation 1 in the manuscript
        self.rSDLogScale1 = sqrt(log(1 + self.CV1 ** 2))
        self.rSDLogScale2 = self.rSDLogScale1

        # the number for pivot, the notation "m" in the manuscript
        nSimulForPivot = 100000-1

        # choosing a seed
        self.seed_value = 12181988

        # Generate 4 set of random numbers each with specified seed, will be used for U1, U2, Z1, and Z2 later (Equation 3)
        np.random.seed(self.seed_value - 1)
        random_numbers1_1 = np.random.rand(nSimulForPivot)

        np.random.seed(self.seed_value - 2)
        random_numbers1_2 = np.random.rand(nSimulForPivot)

        np.random.seed(self.seed_value - 3)
        random_numbers2_1 = np.random.rand(nSimulForPivot)

        np.random.seed(self.seed_value - 4)
        random_numbers2_2 = np.random.rand(nSimulForPivot)

        # Generate random number for later used in calculating Ui and Zi in generalized pivotal method
        # group 1 pivot calculation, U_i and Z_i used in Equation 3
        self.U1 = chi2.ppf(random_numbers1_1, self.N1 - 1 )
        self.Z1 = norm.ppf(random_numbers2_1)

        # group 2 pivot calculation, U_i and Z_i used in Equation 3
        self.U2 = chi2.ppf(random_numbers1_2, self.N2 - 1 )
        self.Z2 = norm.ppf(random_numbers2_2)

    # the main process, method of moments = ['no_moments', 'first_two_moment', 'higher_orders_of_moments']
    def main(self, method_of_moments):
        # the pre-determined list of seeds, using number of nMonte
        list_seeds = [i for i in range(self.seed_value, self.seed_value + self.nMonte)]
        # put the list of seeds into a table (a.k.a DataFrame) with one column named "Seeds"
        df = pd.DataFrame({'Seeds':list_seeds})
        df_record = df.copy()
        #using no method of moments
```

```
        if method_of_moments == 'no_moments':
            # generate log-normal distributed numbers, using mean of rMeanLogScale and standard deviation of rSDLogScale
            df['rSampleOfRandoms'] = df.apply(self.Sample_inv_normal, args=('Seeds',), axis=1)
            # put the table into dask, a progress that can parallel calculating each rows using multi-thread
            df = dd.from_pandas(df['rSampleOfRandoms'], npartitions=35)
            # calculate sample mean and SD using Mean_SD
            df = df.apply(self.Mean_SD, meta=('float64', 'float64'))

        # using method of moments to transform from raw to log mean and SD
        elif method_of_moments in ['first_two_moment', 'higher_orders_of_moments']:
            # generate log-normal distributed numbers, using mean of rMeanLogScale and standard deviation of rSDLogScale, transform values from log to raw with np.exp
            df['rSampleOfRandoms'] = df.apply(self.Sample_inv_normal, args=('Seeds',), axis=1).apply(lambda x: [np.exp(item) for item in x])
            # put the table into dask, a progress that can parallel calculating each rows using multi-thread
            df = dd.from_pandas(df['rSampleOfRandoms'], npartitions=35)
            # calculate sample mean and SD using Mean_SD
            df = df.apply(self.Mean_SD, meta=('float64', 'float64'))

            # using Equation 7 and 8
            if method_of_moments == 'first_two_moment':

                df = df.apply(self.first_two_moment, args=(0,1,2,3), meta=('float64', 'float64')) # generate sample mean and SD in Log scale using Mean_SD (above)

            # using Equation 7 and 9
            elif method_of_moments == 'higher_orders_of_moments':

                df = df.apply(self.higher_orders_of_moments, args=(0,1,2,3), meta=('float64', 'float64'))

        # Equation 3 # generate 'ln_ratio' and 'se_ln_ratio' with sample mean and SD using GPM
        df = df.apply(self.GPM_log_ratio_SD, args=(0,1,2,3), meta=('float64', 'float64'))
        df_record[['ln_ratio', 'se_ln_ratio']] = df.compute().tolist()
        # check coverage of each rows
        df = df.apply(self.Coverage, args=(0,1), meta = ('float64', 'float64'))
        df_record['intervals_include_zero'] = df.compute().tolist()

        # compute the mean of the list of coverage (0 or 1), it equals to the percentage of coverage
        coverage = df.mean().compute()

        return coverage, df_record, self.nMonte, self.N1, self.CV1, method_of_moments

    def Sample_inv_normal(self, row, seed_):
        # using seed from pre-determined list
        np.random.seed(row[seed_])
        # generate log-normal distribution, using mean of rMeanLogScale and standard deviation of rSDLogScale
        rSampleOfRandoms = [(norm.ppf(i,loc=self.rMeanLogScale1, scale=self.rSDLogScale1)) for i in np.random.rand(self.N1+self.N2)]

        return rSampleOfRandoms

    def Mean_SD(self, row):

        rSampleOfRandoms1 = row[:self.N1]
        rSampleOfRandoms2 = row[self.N1:(self.N1+self.N2)]

        # the mean of rSampleOfRandoms1, notation "z_i"
        rSampleMean1 = np.mean(rSampleOfRandoms1)
        # the standard deviation of rSampleOfRandoms1, delta degree of freeden = 1, notation "sz_i"
        rSampleSD1 = np.std(rSampleOfRandoms1, ddof=1)
        rSampleMean2 = np.mean(rSampleOfRandoms2)
        rSampleSD2 = np.std(rSampleOfRandoms2, ddof=1)

        return rSampleMean1, rSampleSD1, rSampleMean2, rSampleSD2

    def first_two_moment(self, row, col_SampleMean1, col_SampleSD1, col_SampleMean2, col_SampleSD2):

        SampleMean1 = row[col_SampleMean1]
        SampleSD1 = row[col_SampleSD1]

        SampleMean2 = row[col_SampleMean2]
        SampleSD2 = row[col_SampleSD2]
```

```
        #using Equation 7 and 8
        rSampleMeanLogScale1, rSampleSDLogScale1, _ = self.transform_from_raw_to_log_mean_SD(SampleMean1, SampleSD1)
        rSampleMeanLogScale2, rSampleSDLogScale2, _ = self.transform_from_raw_to_log_mean_SD(SampleMean2, SampleSD2)

        return rSampleMeanLogScale1, rSampleSDLogScale1, rSampleMeanLogScale2, rSampleSDLogScale2

    def higher_orders_of_moments(self, row, col_SampleMean1, col_SampleSD1, col_SampleMean2, col_SampleSD2):

        SampleMean1 = row[col_SampleMean1]
        SampleSD1 = row[col_SampleSD1]

        SampleMean2 = row[col_SampleMean2]
        SampleSD2 = row[col_SampleSD2]
        #using Equation 7 and 9
        rSampleMeanLogScale1, _, rSampleSDLogScale1 = self.transform_from_raw_to_log_mean_SD(SampleMean1, SampleSD1)
        rSampleMeanLogScale2, _, rSampleSDLogScale2 = self.transform_from_raw_to_log_mean_SD(SampleMean2, SampleSD2)

        return rSampleMeanLogScale1, rSampleSDLogScale1, rSampleMeanLogScale2, rSampleSDLogScale2

    def transform_from_raw_to_log_mean_SD(self, Mean, SD):
        CV = SD/Mean
        CVsq = CV**2
        #Mean in log scale, Equation 7
        MeanLogScale_1 = log(Mean/sqrt(CVsq + 1))
        #SD in log scale, Equation 8
        SDLogScale_1 = sqrt(log((CVsq + 1)))
        #SD in log scale, Equation 9
        SDLogScale_2 = sqrt(CVsq * (1 + CVsq/(1 + CVsq))**2 - (1 + CVsq/(1 + CVsq)) * ((1 + CVsq)**2 - 3 + 2/(1 + CVsq)) + (1/4) * ((1 + CVsq)**4 - 4*(1 + CVsq) - 1 + 8/(1 + CVsq)
- 4/((1 + CVsq)**2)))

        return MeanLogScale_1, SDLogScale_1, SDLogScale_2

    def GPM_log_ratio_SD(self, row, col_SampleMeanLog1, col_SampleSDLog1, col_SampleMeanLog2, col_SampleSDLog2):  # Equation 2 and 3

        #group 1 pivot calculation
        SampleMeanLog1 = row[col_SampleMeanLog1]
        SampleSDLog1 = row[col_SampleSDLog1]
        Pivot1 = self.Pivot_calculation(SampleMeanLog1, SampleSDLog1, self.N1, self.U1, self.Z1)
        #group 2 pivot calculation
        SampleMeanLog2 = row[col_SampleMeanLog2]
        SampleSDLog2 = row[col_SampleSDLog2]
        Pivot2 = self.Pivot_calculation(SampleMeanLog2, SampleSDLog2, self.N2, self.U2, self.Z2)

        # Equation 2, generalized pivotal statistics
        pivot_statistics = np.log(Pivot1) - np.log(Pivot2)

        # Calculate ln ratio and SE ln ratio by percentile and Z statistics, Equation 4 and 5
        ln_ratio = pd.Series(pivot_statistics).quantile(.5)
        se_ln_ratio = (pd.Series(pivot_statistics).quantile(.75) - pd.Series(pivot_statistics).quantile(.25))/(norm.ppf(.75) - norm.ppf(.25))

        return ln_ratio, se_ln_ratio

    def Pivot_calculation(self, rSampleMeanLogScale, rSampleSDLogScale, N, U, Z):
        # Equation 3
        return np.exp(rSampleMeanLogScale- np.sqrt((rSampleSDLogScale**2 * (N-1))/U) * (Z/sqrt(N)) ) * np.sqrt((np.exp((rSampleSDLogScale**2 * (N-1))/U) - 1) *
np.exp((rSampleSDLogScale**2 * (N-1))/U))

    def Coverage(self, row, col_ln_ratio, col_se_ln_ratio):

        ln_ratio = row[col_ln_ratio]
        se_ln_ratio = row[col_se_ln_ratio]

        # Calculate the confidence intervals with z_score of alpha = 0.05, Equation 6
        lower_bound = ln_ratio - self.z_score * se_ln_ratio
        upper_bound = ln_ratio + self.z_score * se_ln_ratio

        intervals_include_zero = (lower_bound < 0) and (upper_bound > 0)
        # 1 as True, 0 as False, check coverage
```

```
        return int(intervals_include_zero)

if __name__ == '__main__':
    # number of Monte Carlo simulations
    nMonteSim = 1000000
    for method_of_moments in ['no_moments', 'first_two_moment', 'higher_orders_of_moments']:
        print(method_of_moments)
        # Sample size, we choose 15, 25, 50, notation "n" in the manuscript
        for N in [15, 25, 50]:
            # coefficient of variation, we choose 0.15, 0.3, 0.5
            for CV in [0.15, 0.3, 0.5]:
                # record the datetime at the start
                start_time = datetime.now()
                print('start_time:', start_time)
                print(f"Start GPM_MC_nMonteSim_{nMonteSim}_N_{N}_CV_{CV}_{str(start_time).split('.')[0].replace('-','').replace(' ','').replace(':','')}_{method_of_moments}")

                # Cal the class SimulPivotMC(), generate variables in the def __init__(self)
                run = SimulPivotMC(nMonteSim, N, CV)
                # start main()
                coverage_by_ln_ratio, df_record, nMonte, N1, CV1, method_of_moments = run.main(method_of_moments=method_of_moments)

                # record the datetime at the end
                end_time = datetime.now()
                # print the datetime at the end
                print('end_time:', end_time)
                # calculate the time taken
                time_difference = end_time - start_time
                print('time_difference:', time_difference)
                # print out the percentage of coverage
                print('percentage coverage: %s' %(coverage_by_ln_ratio,))

                output_txt1 = f"start_time: {start_time}\nend_time: {end_time}\ntime_difference: {time_difference}\n\nnMonte = {nMonte}; N1 = {N1}; CV1 = {CV1}\n\n percentage 
coverage: {coverage_by_ln_ratio}\n"

                output_dir = f"GPM_MC_nMonte_{nMonte}_N_{N1}_CV_{CV1}_{str(end_time).split('.')[0].replace('-','').replace(' ','').replace(':','')}"

                # save the results to the csv
                print('csv save to ' + output_dir + f'_{method_of_moments}.csv')
                df_record.to_csv(output_dir + f'_{method_of_moments}.csv')

                # save the results to the txt
                with open(output_dir + f'_{method_of_moments}.txt', 'w') as f:
                    f.write(output_txt1)
    quit()
```
